# Morphological variation in *Schizothorax oconnori*, *Schizothorax waltoni* (Teleostei: Cyprinidae: Schizothoracinae), and their natural hybrids from the middle Yarlung Zangbo River, Tibet

**DOI:** 10.1002/ece3.11342

**Published:** 2024-05-23

**Authors:** Baoshan Ma, Tianyi Zhao, Bin Xu, Liqiao Zhong, Xiangxiang Wu, Kaijin Wei, Zhiming Zhang, Yunfeng Li

**Affiliations:** ^1^ National Agricultural Science Observing and Experimental Station of Chongqing, Yangtze River Fisheries Research Institute Chinese Academy of Fishery Science Wuhan China; ^2^ Institute of Hydroecology Ministry of Water Resources and Chinese Academy of Sciences Wuhan China

**Keywords:** discriminant function analysis, evolution, morphometric characters, schizothoracine fishes, truss network

## Abstract

The morphological variation in *Schizothorax oconnori*, *Schizothorax waltoni*, and their natural hybrids was examined using conventional and image‐based analysis approaches. In total, 38 specimens of *S. oconnori*, 35 of *S. waltoni*, and 37 natural hybrids were collected from the Shigatse to the Lhasa section of the Yarlung Zangbo River during June and July 2021. A total of 21 morphometric, 4 meristic, and 27 truss variables were employed for the classification of *S. oconnori*, *S. waltoni*, and natural hybrids. Principal component analysis (PCA) and factor analysis (FA), as well as discriminant function analysis (DFA) and cluster analysis (CA), were conducted to identify differences based on traditional and truss measurements. Four principal components explained 75.92% of the variation among the morphometric characters, while five principal components accounted for 79.69% of the variation among the truss distances. FA results showed that factor 1 was associated with head shape, and factor 2 was associated with fins based on morphometric characters. Among the truss characters, factor 1 was related to head shape, and factor 2 was related to chest shape. In DFA, morphometric measurements achieved higher accuracy (100%) compared to truss distances (94.55%). The head morphology of hybrids exhibited intermediate traits between *S. oconnori* and *S. waltoni*. Both morphometry‐based and truss‐based clustering indicated that the morphology of natural hybrids leaned toward *S. oconnori*. In conclusion, the combination of morphometric and truss analysis is beneficial for classifying *S. oconnori*, *S. waltoni*, and their natural hybrids. The presence of natural hybrids could be considered an evolutionary response to the differentiation of nutritional and spatial niches in the middle Yarlung Zangbo River.

## Graphical abstract

The combination of morphometric and truss analysis is beneficial for classifying *Schizothorax oconnori*, *Schizothorax waltoni*, and their natural hybrids. The presence of natural hybrids could be considered an evolutionary response to the differentiation of nutritional and spatial niches in the middle Yarlung Zangbo River.

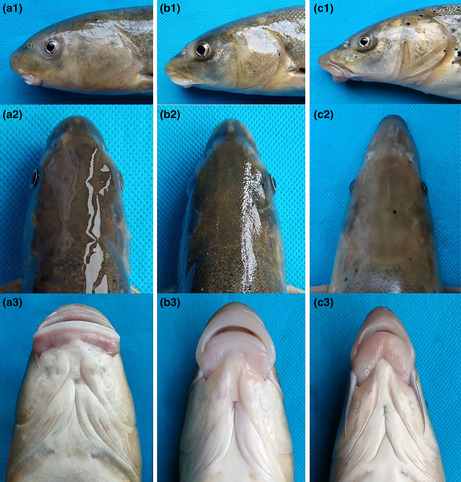

## 1 INTRODUCTION

The development of population conservation strategies and sustainable management plans necessitates thorough research on population structure. Because long‐term evolutionary processes lead to fish hybridization, which alters fish body form and genetic structure, morphometric features are essential for understanding population structure (Ramya et al., 2021; Sajina et al., 2011; Scribner et al., 2000). Traditional morphometry, in contrast to genetic marker‐based approaches, proves to be a cost‐effective and easily operable method. Nevertheless, the use of traditional methods involving morphometric and meristic variables for species identification has recently decreased owing to the scarcity of fish taxonomists and the development of molecular technology. Fortunately, with the development of software technologies, conventional morphometrics incorporated with image analysis are receiving increasing attention (Cadrin & Friedland, 1999; Petrellis, 2021; Sibinamol et al., 2020). The majority of research on species and population discrimination is based on morphological variation in morphometrics and meristics (Sreekanth et al., 2015; Turan, 2004; Wang et al., 2015), truss networks (Rodrigues‐Oliveira et al., 2023; Sajina et al., 2011; Sibinamol et al., 2020), calcified tissues, such as otoliths and skeletons (Cañás et al., 2012; Chen et al., 2021; Vaisakh et al., 2019), and some special tissues, like the exo‐celiac liver (Zhang, 2011).

Conventional morphometric methods have several limitations in depicting the body shape of fish species, principally because the variables are all linear (Ramya et al., 2021; Turan, 1999). The development of image analysis technologies helps to delineate the body shape of fish for species descriptions by applying landmark trusses. Image analysis is extensively employed in interspecific population research for fisheries resource management. It includes a distinctive framework of the entire fish to characterize intraspecific and interspecific morphological alterations within a two‐dimensional outline (Winans, 1984).

Research on the population characterization of endemic species is beneficial for formulating conservation measures (Ramya et al., 2021). Many studies relevant to fish population characterization at low altitudes exist based on morphometry, truss measurements, and molecular markers (Ramya et al., 2021; Wang et al., 2015). However, related research is scarce at high altitudes for endemic species that encounter population degradation. Schizothoracine fishes are endemic to the central Asian plateau, with a total of 99 species and subspecies found in China, some of which hold significant scientific research and economic value (Ma et al., 2023). Regrettably, in recent years, the survival of schizothoracine fishes has been significantly jeopardized by human activities, including hydropower development, overfishing, sand mining, and biological invasion (Ma et al., 2023). Many schizothoracine fishes now face critical endangerment, and are endangered, vulnerable, or near‐threatened (Ma et al., 2023). Notably, *Schizothorax waltoni* has been designated as China's national secondary key protected wildlife due to its declining population numbers (Forestry and Grassland Administration & Ministry of Agriculture and Rural Affairs, 2021). Furthermore, during the long process of evolution, natural hybridization has occurred in several schizothoracine fishes, and research indicates that *S. waltoni* and *Schizothorax oconnori* can produce natural hybrids of each other's parents (Ma et al., 2018). The emergence of natural hybrids with intermediate traits has created challenges in distinguishing these two *Schizothorax* species, and the conservation of endangered fish primarily relies on accurate species identification (Ma et al., 2018).

Natural hybridization plays a crucial role in the evolutionary adaptation of organisms to their environment (Barton, 2001; Seehausen, 2004). Natural hybridization among fish species is common, as noted by Scribner et al. (2000). Instances of such hybridization have been reported in various species, including *Hemibarbus maculatus* and *Hemibarbus labeo* (Xue & Yu, 1959), *Xiphophorus maculatus* and *Xiphophorus helleri* (Powell et al., 2020), *Orestias agassizii* and *Orestias luteus* (Esquer‐Garrigos et al., 2015), and butterfly fishes such as *Chaetodon trifasciatus* and *Chaetodon lunulatus* (Montanari et al., 2012). Natural hybridization can enhance the survival abilities of fishes and their adaptability to changes in habitat (Bittner et al., 2010). It has the potential to create novel multi‐gene complexes, which may lead to hybrid speciation and the emergence of new species (Scribner et al., 2000; Selz et al., 2014). In addition to genetic inheritance, hybrids also exhibit morphological diversity, which is closely linked to niche differentiation (Bittner et al., 2010). Therefore, studying the morphological changes resulting from hybridization between *S. oconnori* and *S. waltoni* in their natural environment holds significant importance for the conservation and management of fish germplasm resources on the Tibetan Plateau.

A comparison of morphological features between the natural hybrid with *S. oconnori* and *S. waltoni* revealed that the hybrid exhibited differences from its parent species in several head traits, displaying typical intermediate characteristics (Ma et al., 2018). Nevertheless, previous studies on natural hybrids with small sample sizes have provided only simple descriptions (Ma et al., 2018; Ren & Ren, 2003) and have not involved comprehensive analyses based on conventional characteristics and truss networks. The complete discrimination of morphological variation among *S. oconnori*, *S. waltoni*, and their natural hybrids has not been achieved. Therefore, research on species identification is imperative to facilitate the sustainable development of conservation and management strategies. Furthermore, the evolutionary status of natural hybrids requires further discussion. In this context, the primary objectives of this study are as follows: (1) to describe the morphological characteristics of *S. oconnori*, *S. waltoni*, and their natural hybrids; (2) to delineate the overall shape of the three populations based on morphometric variations and truss networks; and (3) to investigate the intermediate traits of natural hybrids and explore the relationship between their morphological variation and evolutionary ecology.

## 2 MATERIALS AND METHODS

### 2.1 Sampling

**FIGURE 1:**
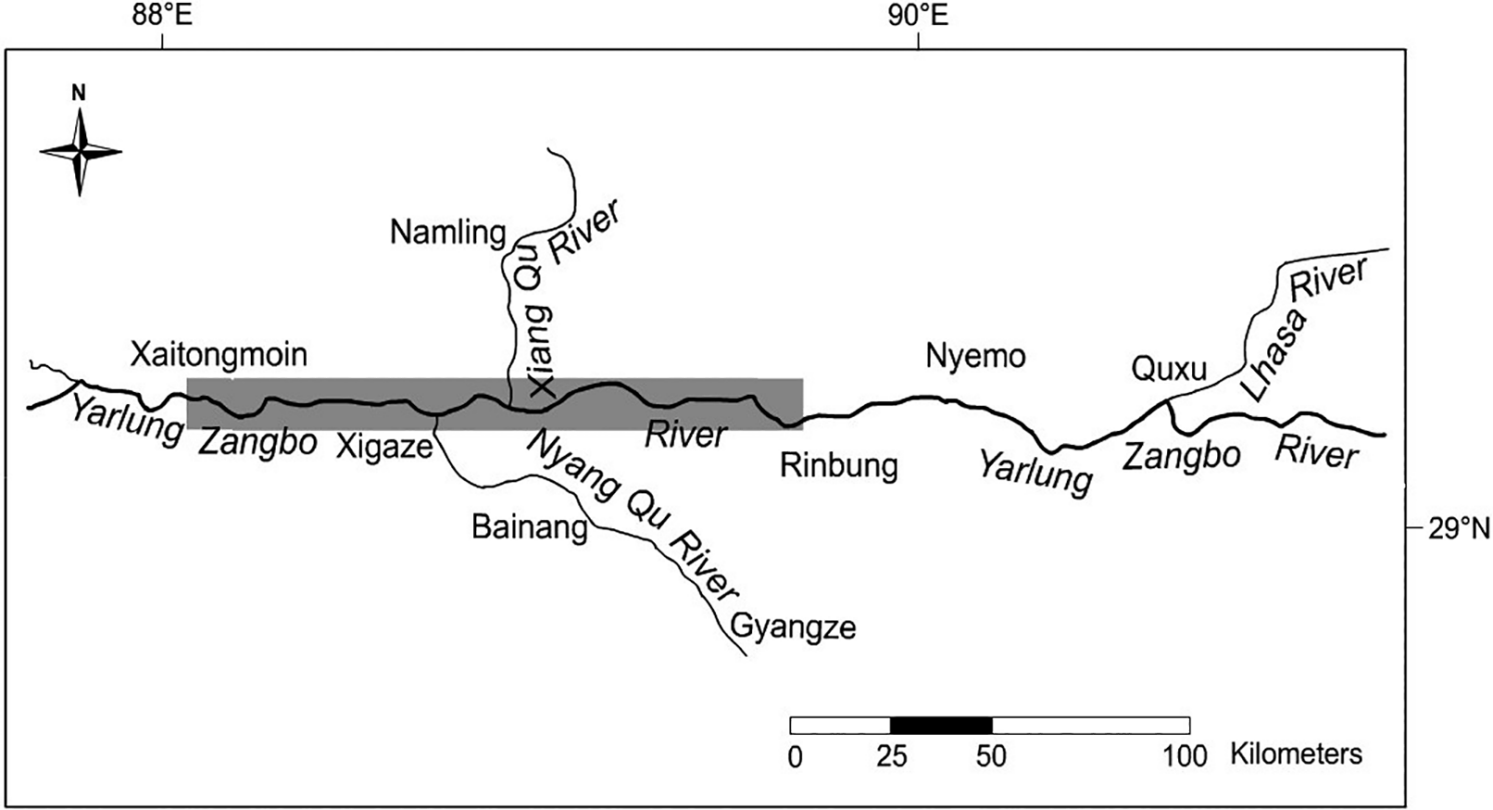
Sampling locations of *Schizothorax oconnori*, *Schizothorax waltoni*, and their natural hybrids from the Shigatse to Lhasa River section of Yarlung Zangbo River during June and July 2021.

Between June and July 2021, a total of 38 specimens of *S. oconnori*, 35 specimens of *S. waltoni*, and 37 natural hybrids were collected from the Shigatse to Lhasa section of the Yarlung Zangbo River (Figure 1). The identification of *S. oconnori* and *S. waltoni* was carried out following the criteria outlined by Chen and Cao (2000), while the identification of natural hybrids was based on the methods established by Ren and Ren (2003) and Ma et al. (2018). Details regarding the standard length and total weight of the specimens are given in Table 1.

**TABLE 1:** Sample information for *Schizothorax oconnori*, *Schizothorax waltoni*, and their natural hybrids.

Species	*n*	Standard length (mm)		Total weight (g)	
		Range	Mean ± SD	Range	Mean ± SD
*S. oconnori*	38	238–432	331.1 ± 40.8	190.4–1406.0	583.7 ± 207.7
*S. waltoni*	35	220–502	363.6 ± 69.5	134.5–1662.0	676.0 ± 378.4
Natural hybrids	37	157–392	286.6 ± 61.6	71.7–812.5	404.6 ± 224.5

### 2.2 Measurement procedure

#### 2.2.1 Morphometric and meristic traits

A total of 21 morphometric variables were measured with precision to the nearest 0.01 mm, using a vernier caliper. Measurements were conducted from the left side of the specimens following standard methods for cyprinid taxonomy, with some adjustments. The morphometric variables are detailed in Table 2. Additionally, four meristic characters were included in the analysis. The process of observing and counting these various characteristics adhered to the methodology outlined by Ramya et al. (2021) and was performed by the same observer twice for consistency.

**TABLE 2:** Morphometric characters of *Schizothorax oconnori*, *Schizothorax waltoni*, and their natural hybrids.

No.	Code	Morphometric characters	Definitions
1	SL	Standard length	The straight‐line measurement from anterior end of snout to base of caudal fin
2	TL	Total length	The straight‐line measurement from anterior end of snout to tip of caudal fin
3	FL	Fork length	The straight‐line measurement from anterior end of snout to fork of caudal fin
4	BD	Body depth	The straight‐line measurement between dorsal and ventral surfaces at the point of origin of dorsal fin
5	BW	Body width	The horizontal‐line measurement at the point of origin of dorsal fin
6	CPL	Length of caudal peduncle	The straight‐line measurement taken from the base of the anal fin to the base of the caudal fin
7	CPD	Depth of caudal peduncle	The straight‐line measurement at the minimum depth from dorsal to ventral surface of the caudal peduncle
8	LDF	Length of dorsal fin	Measurement from proximal to distal end of dorsal fin
9	LPF	Length of pectoral fin	Measurement from proximal to distal end of pectoral fin
10	LVF	Length of ventral fin	Measurement from proximal to distal end of pelvic fin
11	LAF	Length of anal fin	Measurement from proximal to distal end of anal fin
12	HL	Head length	Measurement from anterior end of snout to posterior edge of opercular bone
13	HD	Head depth	The straight‐line measurement of head at posterior margin of opercular bone
14	HW	Head width	The horizontal‐line measurement of head at posterior margin of opercular bone
15	SnL	Snout length	The straight‐line measurement from anterior end of snout to anterior end of eye orbit
16	OFW	Width of oral fissure	The horizontal‐line measurement between the joint of upper and lower jaw on left and right sides
17	OFD	Depth of oral fissure	The straight‐line measurement between upper and lower jaw when mouth is opened to maximum
18	SWL	Length of snout whisker	The straight‐line measurement of snout whiskers
19	MWL	Length of maxillary whisker	The straight‐line measurement of maxillary whiskers
20	ED	Eye diameter	The straight‐line measurement from anterior to posterior end of eye orbit
21	ID	Interorbital distance	The horizontal distance between upper edges of the eyes on the back of the head

#### 2.2.2 Truss network

**FIGURE 2:**
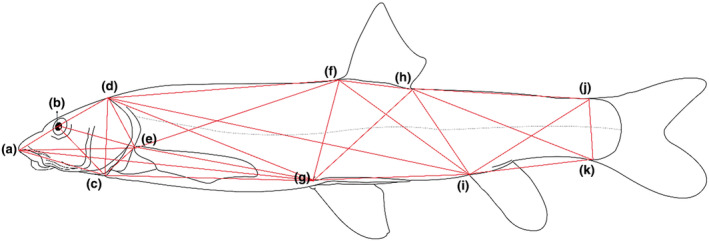
Landmarks used for truss analysis: (a) anterior tip of the snout on upper jaw, (b) center of eye, (c) ventral insertion of opercular bone, (d) nape above insertion of opercular bone, (e) origin of pectoral fin, (f) origin of dorsal fin, (g) origin of pelvic fin, (h) end of dorsal fin base, (i) origin of anal fin, (j) dorsal origin of caudal fin, (k) ventral origin of caudal fin.

The fresh specimens were positioned on a blue background plate marked with a scale, keeping the left side up. Additionally, the fins were secured in place using pins (Ramya et al., 2021). Each specimen was assigned a unique code and number corresponding to their respective populations to facilitate future tracking. A digital camera (Canon EOS M6, Japan) was used to capture the images. Photographs, including landmarks, are displayed in Figure 2.

In total, 11 landmarks were employed to construct a truss network. The truss network analysis, referred to as geomorphometric analysis, was conducted by connecting various landmarks to each other, forming quadrilaterals that represent the morphology of each specimen. In total, each specimen had a trussed box comprised of 27 lines, connecting these 11 landmarks and outlining its fundamental body shape (Strauss & Bookstein, 1982). The landmarks were digitized, and the distances between the landmarks were measured directly on the images using Image J software. Subsequently, all the distance values were exported to a spreadsheet.

### 2.3 Data analysis

To eliminate the length effect, the 20 morphometric characters and 27 truss distances were normalized by dividing them by the standard length. The standard length itself was not utilized in subsequent analyses. The efficacy of this transformation was evaluated by calculating correlation coefficients between the variables and the standard length (Turan, 1999). For the purpose of detecting significant differences among *S. oconnori*, *S. waltoni*, and their natural hybrids in all measurements (including morphometric, meristic, and truss data), univariate ANOVA was conducted. Subsequently, Tukey's post hoc test was employed to further explore specific differences among these populations.

The transformed morphometric characters and truss distances were subjected to principal component analysis (PCA). This analytical approach not only reduces the dimensionality of the data but also helps to minimize redundancy among the specimens. Principal components (PCs) were applied to generate models and extract the principal loadings for further analysis (Wang et al., 2015). Factor analysis (FA) was employed to consolidate the data into several dimensions by condensing a large number of variables into a smaller set of potential variables (Ramya et al., 2021). In FA, factors were determined based on the Guttman‐Kaiser rule, with eigenvalue >1. Subsequently, maximum variable rotation was conducted on these factors to extract variables with high loadings.

Discriminant function analysis (DFA) was utilized to assess the accuracy of classifying individuals within each population and to calculate the success rates of such classification. DFA serves as a predictive model for group membership. In this analysis, the stepwise method was employed to identify key characters with significant influence (Ramya et al., 2021). CA was employed to analyze the multivariate data, with the objective of grouping similar populations into common clusters. Hierarchical cluster analysis was performed based on the Bray–Curtis similarity of morphometric measurements and truss distances among populations.

For the statistical analyses, univariate ANOVA was carried out using SPSS 22.0. Multivariate analyses, including PCA, were conducted using Origin 2022, while FA and DFA were performed using SPSS 22.0. CA was performed using Primer 6.0 software.

## 3 RESULTS

### 3.1 Morphometric and truss characteristics

**FIGURE 3:**
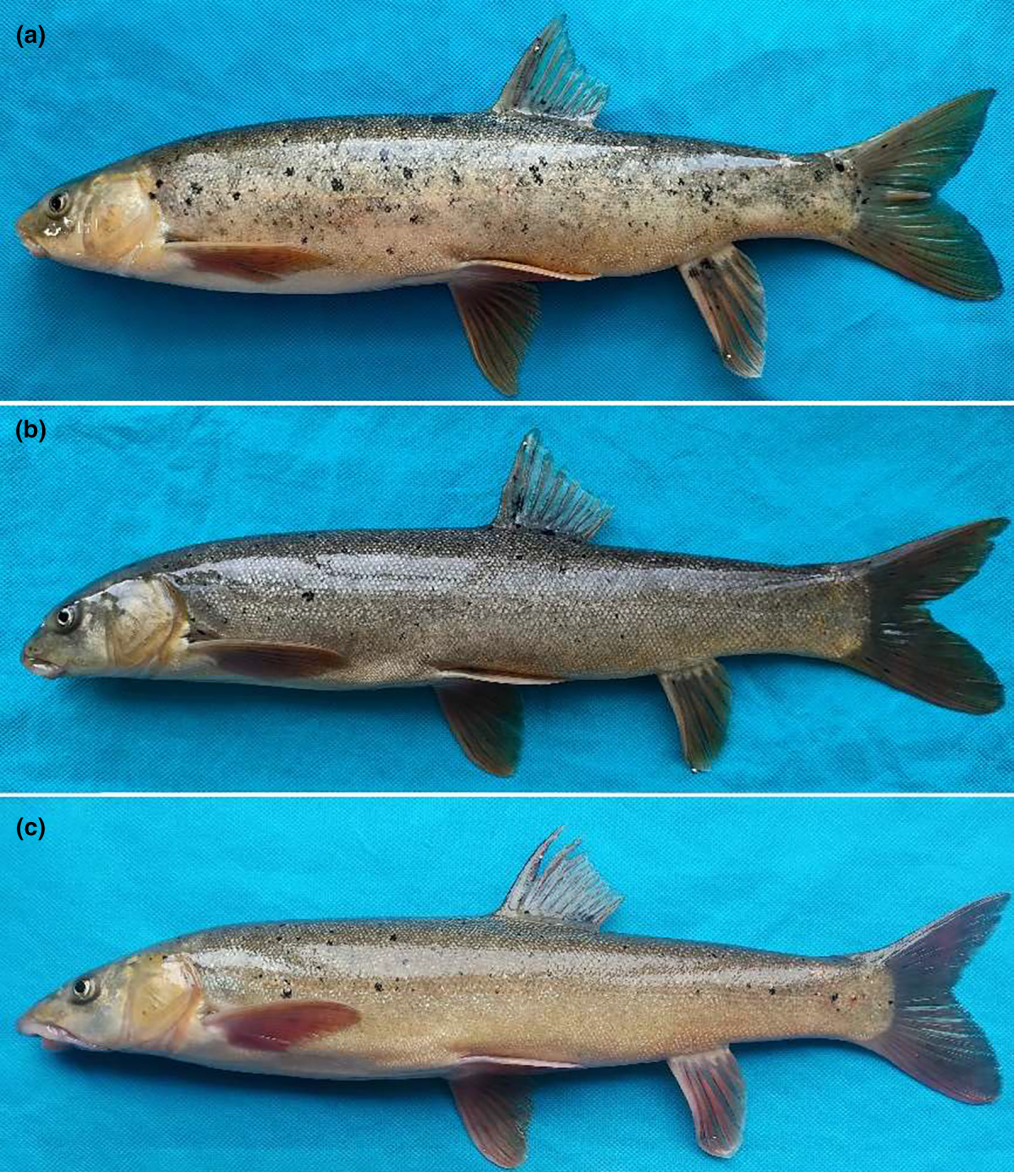
External morphology of *Schizothorax oconnori*, *Schizothorax waltoni*, and their natural hybrid. (a) *S. oconnori*; (b) natural hybrid; (c) *S. waltoni*.

**FIGURE 4:**
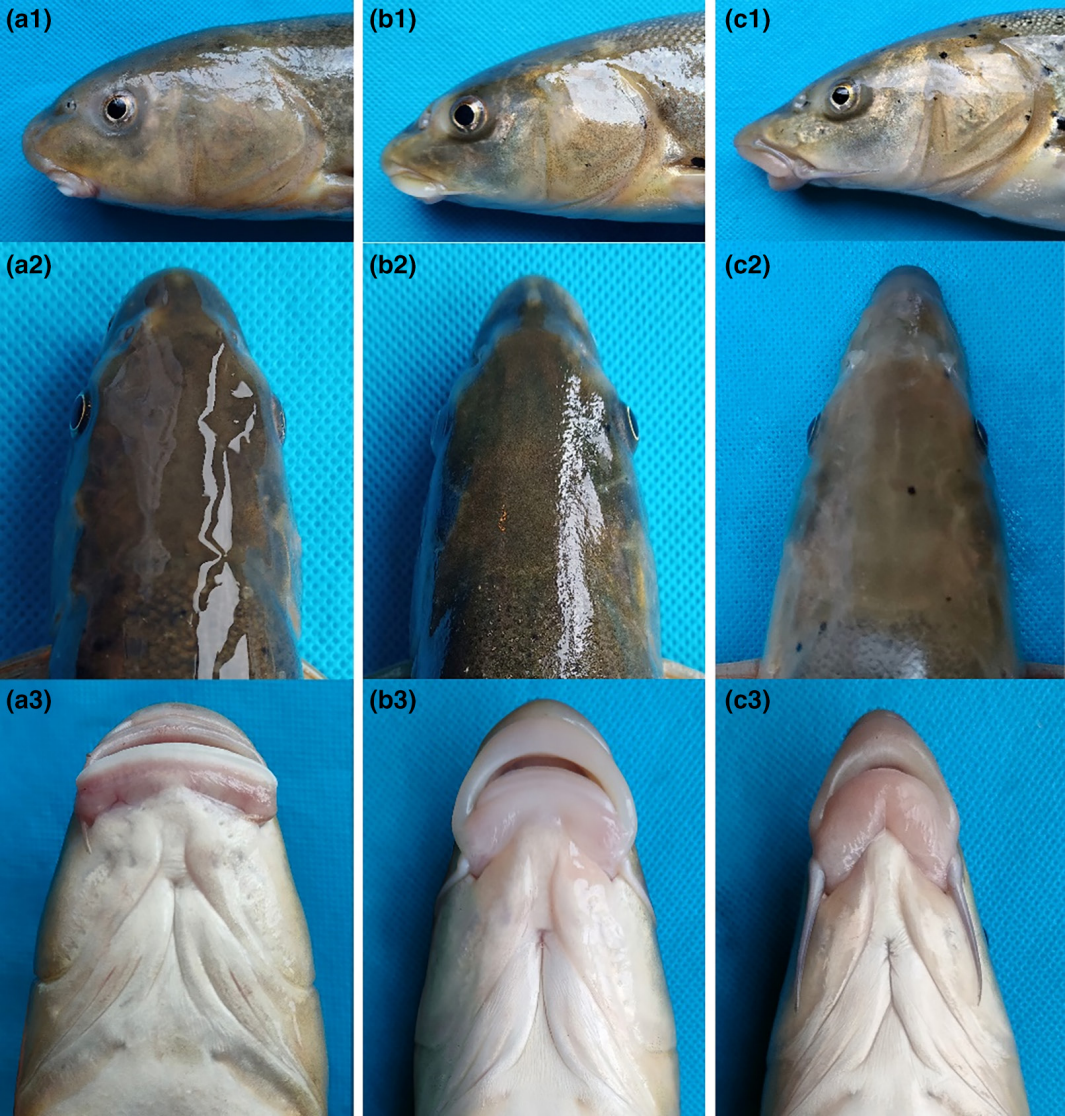
Head morphology of *Schizothorax oconnori*, *Schizothorax waltoni*, and their natural hybrid. (a) *S. oconnori*; (b) natural hybrid; (c) *S. waltoni*; 1. left side of head; 2. dorsal side of head; 3. ventral side of head.

In *S. oconnori*, the snout displayed a round and blunt shape, while the mouth was straight with a sharp and horny lower lip. The maxillary whiskers were relatively short. *S. waltoni* exhibited a cuspidal snout, a horseshoe‐shaped mouth, and a thickly furred lower lip, with notably long maxillary whiskers. The natural hybrids displayed typical intermediate characteristics, positioned between those of the two aforementioned fish species. They had medium‐length maxillary whiskers and lacked obvious skin folds or cuticles in the lower lip (Figures 3 and 4).

Regarding the four meristic characters in *S. oconnori*, *S. waltoni*, and their natural hybrids, significant differences were observed only in pectoral fin rays among the three populations (ANOVA, *p* < .05). There were no distinctions in dorsal fin rays, pelvic fin rays, or anal fin rays among these populations. Further details are provided in Table 3.

**TABLE 3:** Meristic characters for *Schizothorax oconnori*, *Schizothorax waltoni*, and their natural hybrids.

Characters	Code	*S. oconnori*	*S. waltoni*	Natural hybrids	*p* Value
Dorsal fin rays	DFN	7–9	7–9	8	.879
Pectoral fin rays	PFN	14–17	14–18	15–17	.000
Pelvic fin rays	PLN	8–10	8–10	8–9	.951
Anal fin rays	AFN	5	5	5	

After transformation, the correlation coefficient of 20 size‐independent morphometric characteristics with standard length decreased sharply. The correlation coefficients of the primary data ranged from 0.435 to 0.999, while 90% of the transformed values exhibited correlations less than 0.6 after adjustment. Among the captured specimens, *S. waltoni* exhibited the greatest length, followed by *S. oconnori*, and the natural hybrids. The standard lengths were 363.6 ± 69.5 mm for *S. waltoni*, 331.1 ± 40.8 mm for *S. oconnori*, and 286.6 ± 61.6 mm for the natural hybrids. The ratio between the specimen number and the morphometric variables (N:P) was 5.5 for subsequent multivariate analysis.

One‐way ANOVA test revealed significant differences in 19 morphometric characters among *S. oconnori*, *S. waltoni*, and their natural hybrids (*p* < .05). Only the difference in HD among the three populations did not reach a significant level (*p* = .078). The CPL, HL, SnL, OFW, OFD, SWL, MWL, and ID of the natural hybrids were between the values of *S. oconnori* and *S. waltoni*. Additionally, the measurements of FL, BD, CPD, LAF, and HW in the natural hybrids and *S. oconnori* were significantly higher than those in *S. waltoni*. Furthermore, the highest values of TL, BW, LDF, and LVF were observed in the natural hybrids, followed by *S. oconnori*, with *S. waltoni* exhibiting the lowest values (*p* < .05, Table 4).

**TABLE 4:** ANOVA table for morphometric measurements of *Schizothorax oconnori*, *Schizothorax waltoni*, and their natural hybrids.

Code	*S. oconnori*	Natural hybrid	*S. waltoni*
TL	1.190 ± 0.017^b^	1.200 ± 0.023^a^	1.177 ± 0.012^c^
FL	1.096 ± 0.008^a^	1.095 ± 0.017^a^	1.088 ± 0.009^b^
BD	0.205 ± 0.014^a^	0.208 ± 0.018^a^	0.187 ± 0.010^b^
BW	0.157 ± 0.012^b^	0.164 ± 0.016^a^	0.149 ± 0.008^c^
CPL	0.157 ± 0.012^c^	0.164 ± 0.009^b^	0.172 ± 0.011^a^
CPD	0.105 ± 0.005^a^	0.105 ± 0.008^a^	0.097 ± 0.004^b^
LDF	0.134 ± 0.011^b^	0.145 ± 0.016^a^	0.126 ± 0.015^c^
LPF	0.175 ± 0.012^ab^	0.181 ± 0.010^a^	0.170 ± 0.015^b^
LVF	0.166 ± 0.009^b^	0.173 ± 0.010^a^	0.159 ± 0.016^b^
LAF	0.150 ± 0.011^a^	0.149 ± 0.013^a^	0.141 ± 0.010^b^
HL	0.191 ± 0.009^c^	0.208 ± 0.010^b^	0.235 ± 0.010^a^
HD	0.160 ± 0.009	0.163 ± 0.011	0.158 ± 0.008
HW	0.141 ± 0.008^a^	0.140 ± 0.009^a^	0.133 ± 0.006^b^
SnL	0.070 ± 0.006^c^	0.074 ± 0.005^b^	0.093 ± 0.006^a^
OFW	0.076 ± 0.007^a^	0.063 ± 0.005^b^	0.056 ± 0.005^c^
OFD	0.059 ± 0.007^c^	0.066 ± 0.005^b^	0.092 ± 0.012^a^
SWL	0.018 ± 0.003^c^	0.034 ± 0.004^b^	0.051 ± 0.007^a^
MWL	0.023 ± 0.004^c^	0.043 ± 0.005^b^	0.067 ± 0.008^a^
ED	0.029 ± 0.003^b^	0.033 ± 0.005^a^	0.029 ± 0.004^b^
ID	0.097 ± 0.005^a^	0.092 ± 0.006^b^	0.084 ± 0.005^c^

All of the truss distances displayed correlations of less than 0.6 with the standard length, indicating the successful elimination of size effects after transformation. The ANOVA results indicated significant differences (*p* < .01) among the three fish populations in the 25 truss distances analyzed in the study, except for a‐g and b‐c (*p* > .05, see Table 5). Consequently, a‐g and b‐c were excluded from further analysis. The ratio between the specimen number and significant truss distance (N:P) was 4.4 for subsequent multivariate analysis.

**TABLE 5:** ANOVA table for truss distances of *Schizothorax oconnori*, *Schizothorax waltoni*, and their natural hybrids.

Code	*F*	*p*
Distance (a‐b)	60.078	.000
Distance (a‐c)	40.671	.000
Distance (a‐e)	56.636	.000
Distance (a‐g)	0.968	.383
Distance (b‐c)	2.219	.114
Distance (b‐d)	17.081	.000
Distance (b‐e)	16.407	.000
Distance (c‐d)	5.627	.005
Distance (c‐e)	10.350	.000
Distance (c‐g)	92.501	.000
Distance (d‐e)	8.352	.000
Distance (d‐f)	56.883	.000
Distance (d‐g)	42.752	.000
Distance (d‐i)	53.371	.000
Distance (e‐f)	110.151	.000
Distance (e‐g)	99.477	.000
Distance (f‐g)	13.519	.000
Distance (f‐h)	16.830	.000
Distance (f‐i)	17.228	.000
Distance (g‐h)	16.513	.000
Distance (g‐i)	7.563	.001
Distance (h‐i)	10.519	.000
Distance (h‐j)	33.083	.000
Distance (h‐k)	17.161	.000
Distance (i‐j)	22.067	.000
Distance (i‐k)	25.178	.000
Distance (j‐k)	15.862	.000

### 3.2 Discrimination by PCA and FA

**FIGURE 5:**
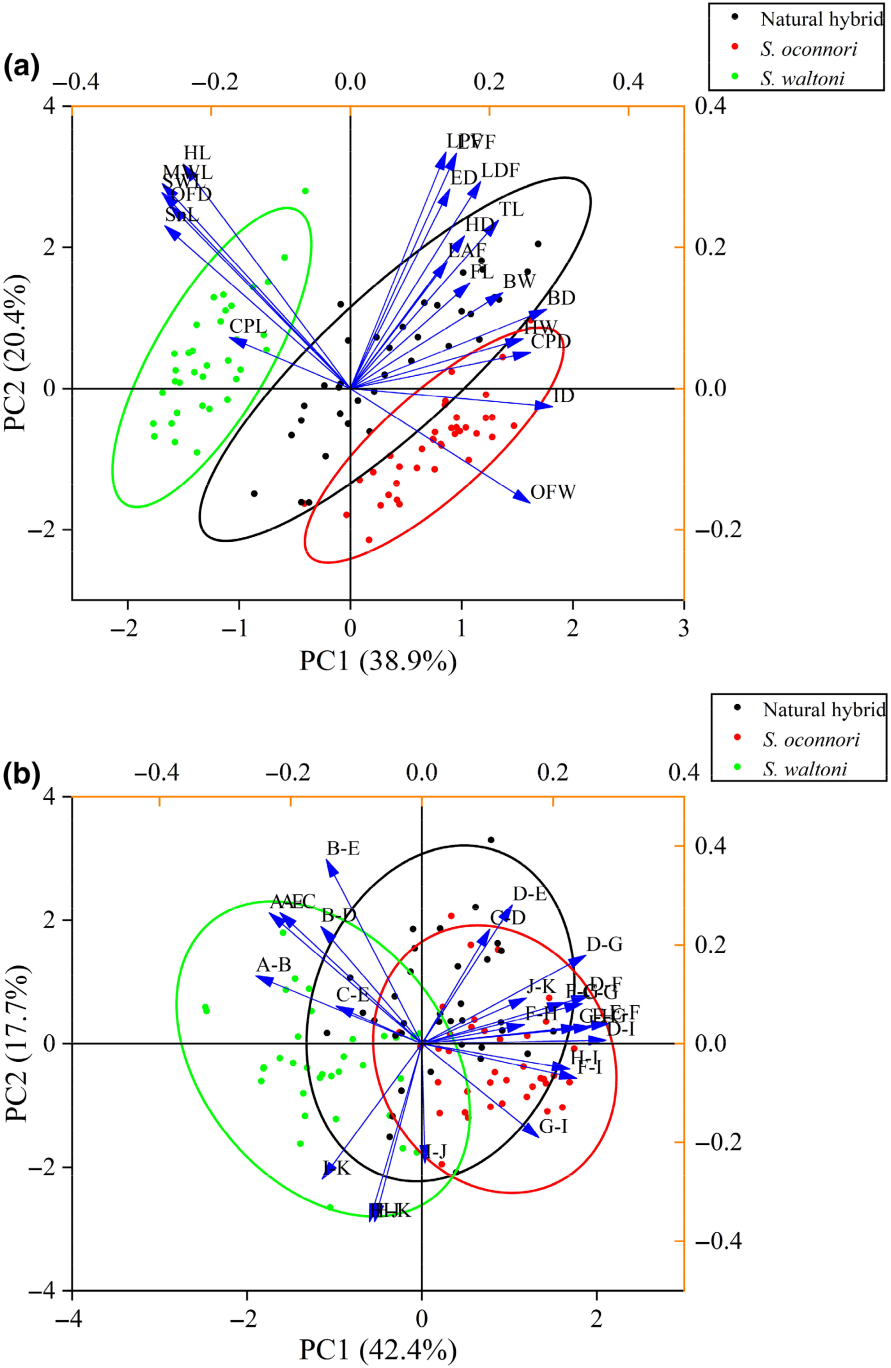
PCA biplot between stocks based on morphometric measurements (a) and truss distances (b). The ellipse design covers 95% of the variability of data. The arrows represent the morphometry measurements.

According to the PCA, four components with eigenvalues >1 were extracted from the morphometric measurements that explained 75.61% of the variation among the three populations. The first and second components explained 38.86% and 20.40% of the variation, respectively. The principal components, variables, and their respective loadings are displayed in Table 6 and Table S1. The biplot representation indicated a clear separation of *S. waltoni* from the other two populations, whereas *S. oconnori* and the natural hybrids exhibited some overlap (Figure 5a). In terms of the variables, those associated with head shape demonstrated predominantly negative correlations with PCA axis 1, whereas variables related to fins displayed positive correlations with axis 2.

**TABLE 6:** Loading of each component in PCA based on morphometric characters.

Code	Component			
	1	2	3	4
TL	0.594	0.481	−0.226	0.073
FL	0.478	0.302	−0.100	0.383
BD	0.787	0.227	0.463	−0.135
BW	0.611	0.274	0.598	−0.18
CPL	−0.486	0.146	0.276	−0.232
CPD	0.723	0.102	0.200	0.056
LDF	0.522	0.592	−0.301	−0.321
LPF	0.383	0.676	−0.292	0.101
LVF	0.425	0.673	−0.389	−0.093
LAF	0.388	0.363	−0.449	0.252
HL	−0.670	0.642	0.073	0.161
HD	0.458	0.437	0.591	−0.001
HW	0.693	0.142	0.567	0.102
SnL	−0.744	0.465	0.132	0.352
OFW	0.721	−0.329	−0.031	0.452
OFD	−0.722	0.523	0.19	0.251
SWL	−0.756	0.561	0.176	0.012
MWL	−0.754	0.586	0.187	0.033
ED	0.398	0.571	−0.263	−0.39
ID	0.811	−0.053	0.088	0.310

The first five principal components with eigenvalues >1 were extracted from truss distances and explained 79.69% of the variation among the various populations. PC1 exhibited the most variation (42.35%) among the samples, followed by PC2 and PC3, which explained 17.68% and 9.01%, respectively. The principal components and variables, along with their loadings, are presented in Table 7 and Table S2. The biplot illustrated a distinct separation of *S. waltoni* from *S. oconnori*, while the natural hybrids displayed some overlap with both *S. waltoni* and *S. oconnori* (Figure 5b). The landmarks associated with the head shape exhibited primarily negative correlations with PCA axis 1, while the landmarks linked to the chest shape displayed positive correlations with PCA axis 2.

**TABLE 7:** Loading of each component in PCA based on truss distances.

Code	Component			
	1	2	3	4
Distance (a‐b)	−0.825	0.289	0.143	0.065
Distance (a‐c)	−0.705	0.558	0.205	0.112
Distance (a‐e)	−0.758	0.557	0.146	0.174
Distance (b‐d)	−0.502	0.499	0.381	0.043
Distance (b‐e)	−0.475	0.784	0.138	0.187
Distance (c‐d)	0.337	0.488	0.639	−0.06
Distance (c‐e)	−0.426	0.161	0.259	0.179
Distance (c‐g)	0.796	0.171	−0.131	−0.106
Distance (d‐e)	0.449	0.591	0.285	−0.063
Distance (d‐f)	0.829	0.205	−0.213	0.144
Distance (d‐g)	0.814	0.376	0.005	−0.027
Distance (d‐i)	0.914	0.015	−0.08	0.247
Distance (e‐f)	0.928	0.085	−0.118	0.000
Distance (e‐g)	0.841	0.071	−0.1	−0.147
Distance (f‐g)	0.71	0.175	0.38	0.089
Distance (f‐h)	0.509	0.081	0.192	−0.571
Distance (f‐i)	0.769	−0.148	0.344	0.267
Distance (g‐h)	0.779	0.07	0.241	−0.036
Distance (g‐i)	0.58	−0.4	−0.009	0.392
Distance (h‐i)	0.733	−0.108	0.37	0.402
Distance (h‐j)	−0.261	−0.758	0.371	0.227
Distance (h‐k)	−0.235	−0.757	0.371	0.242
Distance (i‐j)	0.015	−0.508	0.665	−0.344
Distance (i‐k)	−0.495	−0.576	0.244	−0.357
Distance (j‐k)	0.519	0.196	0.285	−0.277

Factor analysis of the morphometric measurements revealed 65.89% of the variation in the first three factors, of which factor 1 explained 26.92% of the variation and factor 2 and factor 3 explained 19.49% and 19.48%, respectively. According to the morphometric characteristics, factor 1 with the highest loadings was MWL, OFD, HL, SnL, SWL, OFW, and ID. Factor 2 explained variations mainly in the LVF, LDF, ED, LPF, TL, and LAF. BW, BD, HW, HD, and CPD had the highest loadings on factor 3. The morphometric characteristics of factor 1 were concentrated in the heads of the three populations. The characteristics of factor 2 were mainly associated with fins, while the characteristics of factor 3 were mainly related to several variables associated with swimming and locomotion ability.

Three factors were extracted from factor analysis of truss distances among the different populations, which revealed 53.96% of the variation with an eigenvalue >1. Factor 1 explained 20.47%, and factor 2 and factor 3 explained 18.54% and 14.95% of the variation, respectively. Factor 1 with high loading in the truss distance were a‐e, b‐e, a‐c, b‐d, a‐b, g‐i, c‐e, e‐f, d‐i, and c‐d. Factor 2 elucidated the truss distances such as c‐g, e‐g, d‐g, e‐f, d‐f, d‐i, and h‐i. Factor 1 emphasized the most variations in head shape, and factor 2 was related to chest shape.

### 3.3 Discrimination by DFA and CA

**FIGURE 6:**
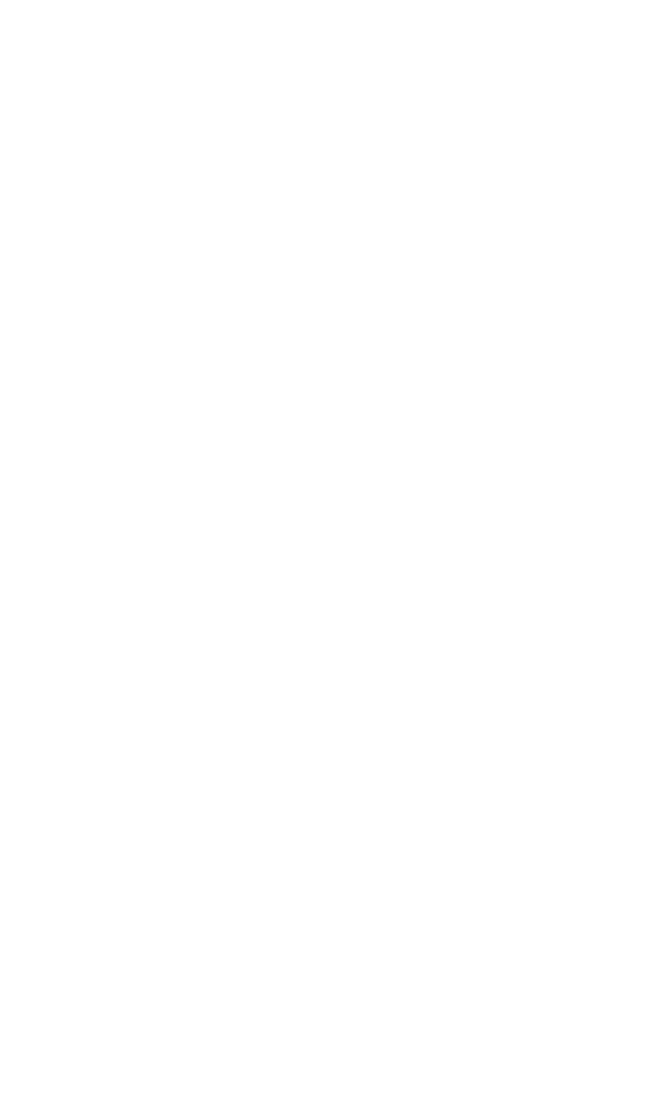
Discriminate function plot from morphometric measurements (a) and truss distances (b).

The first discriminant function was associated with 96.3% of the morphometric characteristics, followed by the second discriminant function, with a value of 3.7%. The discriminant function plot clearly demonstrated the separation of the three populations from each other. Moreover, compared to *S. waltoni*, the natural hybrid appeared to be closer in proximity to *S. oconnori*. Discriminant analysis revealed that 100% of the individuals in all the populations were correctly classified (Figure 6a). The MWL, OFW, OFD, LVF, SnL, ID, and LAF characteristics had maximum loadings, with major effects on population discrimination.

Based on the truss distances, the percentages of separations achieved by the first and second discriminate functions were 79.4% and 20.6%, respectively. A discriminative function plot showed that the three populations were separated from each other (Figure 6b). Discriminant analysis revealed that 94.3% of the *S. oconnori* individuals, 94.7% of the *S. waltoni* individuals, and 94.6% of the natural hybrids were correctly classified (Figure 6b), for a total discriminant success rate of 94.55%. The distances of e‐f, i‐j, f‐g, d‐f, d‐e, a‐c, j‐k, h‐j, and c‐d displayed maximum loadings and exerted a significant influence on the discrimination of populations.

**FIGURE 7:**
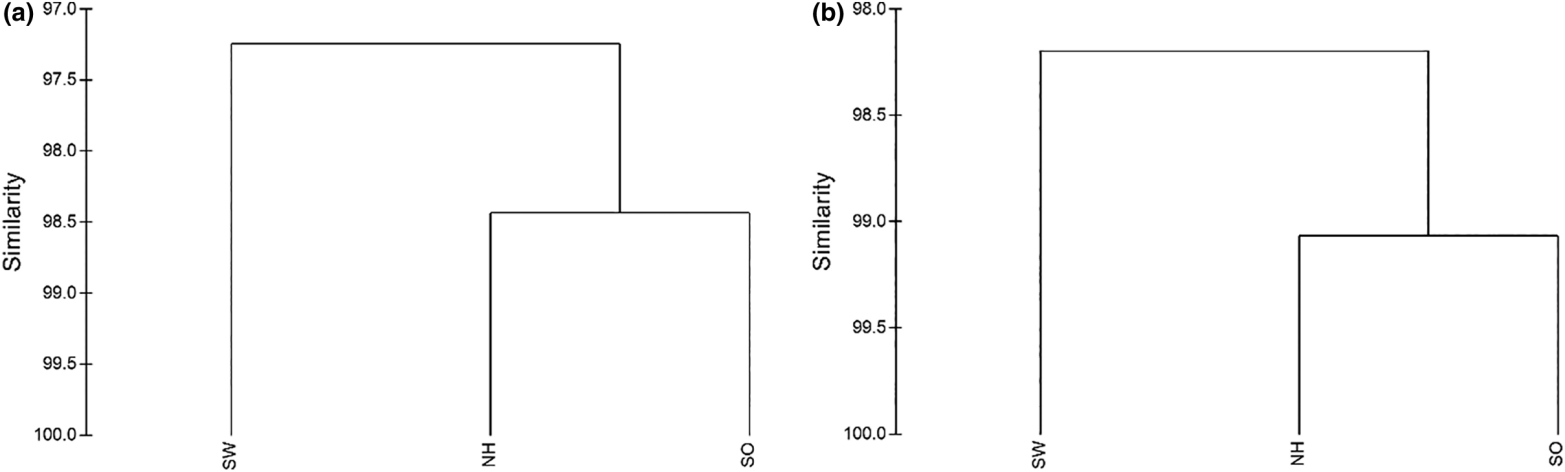
Dendrogram from morphometric measurements (a) and truss distances (b). Population: NY, natural hybrid; SO, *Schizothorax oconnori*; SW, *Schizothorax waltoni*.

Hierarchical cluster analysis was conducted using the Bray–Curtis similarity of morphometric measurements and truss distances among the three populations. The dendrograms derived from both morphometric measurements and truss distances indicated that *S. waltoni* distinguished itself from the other two populations, forming a distinct cluster, while *S. oconnori* and the natural hybrids were grouped together in another cluster (Figure 7).

## 4 DISCUSSION

### 4.1 Advantages of combining morphometric and truss analysis

Species identification is a fundamental requirement for effective management of natural fish populations. In this study, we employed traditional methods involving morphometric and meristic characters, as well as the truss network method utilizing landmarks, to distinguish *S. oconnori*, *S. waltoni*, and their natural hybrids. It is worth noting that a sufficient sample size with a minimum N:P ratio of 3–3.5 is crucial for drawing meaningful conclusions through multivariate analysis (Kocovsky et al., 2009). Our ratios were 5.5 and 4.4 for morphometric measurements and truss distances, respectively, meeting the requirements. Similar ratios of 3.5 and 5.4 were detected for the stock structure of *Barbodes carnaticus* (Ramya et al., 2021).

This study has provided insights into the variations among populations through both conventional and image‐based morphometric analyses. Recent research has also employed morphometric measurements and truss distances for population and species identification in various species, including *Megalaspis cordyla* (Vaisakh et al., 2019), *B. carnaticus* (Ramya et al., 2021), and species of tilapia (Fatsi et al., 2021). Nie et al. (2014) conducted a similar analysis for species validation of six schizothoracine fishes in the Muzhazi River using multivariate analysis.

The PCA provided a model for grouping each fish into a particular population; in our investigation, morphometric factors showed more distinct variation than did truss distances. DFA has also proven to be an effective method for distinguishing between different populations and remains one of the best approaches for group discrimination based on phenotypic characteristics (Palma & Andrade, 2002; Ramya et al., 2021). In our current research, DFA applied to traditional morphometry achieved a high level of accuracy (100%), surpassing the accuracy achieved with truss distances (94.55%). Therefore, it can be concluded that morphometric measurements alone are sufficient for discriminating *S. oconnori*, *S. waltoni*, and their natural hybrids. Additionally, the accuracy of classification can be further improved by incorporating advanced truss distance data, a result consistent with the observations in *B. carnaticus* by Ramya et al. (2021).

The major advantage of conventional methods incorporating truss network analysis for species delineation is the ability to identify various species and determine interspecies differentiation. In contrast to genetic marker‐based methods, morphological approaches are less labor‐intensive and require expertise only in data processing and analysis (Ramya et al., 2021). Since schizothoracine fishes are polyploid fishes (Ma et al., 2023), identifying their hybrids by molecular markers is difficult. Therefore, morphology methods are effective for identifying *S. oconnori*, *S. waltoni*, and their natural hybrids. Nevertheless, there was little difference in the meristic characteristics among the populations in this study. *Nemipterus japonicus* and *B. carnaticus* stock structures also showed a similar phenomenon, so meristic features were not the cause of the difference between populations (Ramya et al., 2021; Sreekanth et al., 2013).

### 4.2 Intermediate traits of natural hybrids

In this research, eight measurements, specifically CPL, HL, SnL, OFW, OFD, SWL, MWL, and ID, in the natural hybrids were observed to fall between the measurements of *S. oconnori* and *S. waltoni*, aligning with the descriptions provided by Ren and Ren (2003) and Ma et al. (2018). Similar outcomes have been reported in studies of other fish species. The natural hybrids of *Hemibarbus maculatus* and *H. labeo* were between their parents in terms of snout shape, snout length, and gill rake number (Xue & Yu, 1959). The HL/SL, ID/ED, ID/HL, SWL/HL, and MWL/HL ratios in the offspring of *Schizothorax wangchiachii* and *Percocypris pingi* differ significantly from those of their parents, with typical intermediate characteristics (Gu et al., 2019). The above phenomenon revealed that differences in head shape may exist in natural hybrids, and the head morphology of hybrids usually differs between their parents.

In this study, both morphometry‐based and truss‐based cluster analyses consistently indicated that the natural hybrids displayed greater similarity to *S. oconnori* than to *S. waltoni*. Notably, five measurements (FL, BD, CPD, LAF, and HW) of the natural hybrids were near *S. oconnori* but significantly greater than those observed for *S. waltoni*. Additionally, the condition factor of the natural hybrids was similar to that of *S. oconnori* but much greater than that of *S. waltoni* (unpublished data). Similarly, our unpublished breeding data revealed that the growth rate of hybrid offspring closely resembled that of *S. oconnori* but was considerably faster than that of *S. waltoni*. Previous research has demonstrated that *S. oconnori* and *S. waltoni* can produce natural hybrids, with a higher incidence of natural hybrids having *S. oconnori* as the female parent (Ma et al., 2018). After maternal inheritance, the expression of mitochondrial genes might affect an organism's outward morphological features (Bolnick et al., 2008). Consequently, the external morphological traits of hybrid offspring typically lean toward those of their mothers (Li et al., 2019). This may account for the observed bias in the morphology of the natural hybrids toward *S. oconnori*. Further confirmation of this phenomenon can be achieved by examining a combination of mitochondrial and cytonuclear genes.

### 4.3 Morphological variation and evolutionary ecology

Morphological characteristics serve as valuable evidence for understanding essential ecological aspects, revealing the strategies employed by organisms and their adaptations to the environment. Moreover, they constitute the foundation for predicting niche relationships (Granier et al., 2006; Wang et al., 2015). Many morphological variables are closely linked to functional traits and nutritional differentiation in river fishes (Acar & Kaymak, 2023; Scharnweber, 2020; Zhang et al., 2020). In our study, *S. oconnori*, *S. waltoni*, and the natural hybrid species displayed substantial variations in head shape, fin length, and chest shape. Variability within the head and snout regions could be associated with feeding patterns and habitat utilization, while variations in fin length may be linked to swimming ability, in line with findings from Nie et al. (2014) and Wang et al. (2015). The observed differences and alterations in head traits and chest shape among the three populations align with results obtained from studies on other schizothoracine fishes, including *Schizothorax kozlovi*, *Schizothorax graham*, and *Schizothorax lissolabiatus* (Lin et al., 2010), *S. kozlovi* and *Schizothorax davidi* (Li et al., 2015), and *Schizothorax biddulphi* and *Schizothorax irregularis* (Yang et al., 2018).

*Schizothorax oconnori* and *S. waltoni* are endemic to the Yarlung Zangbo River in Tibet, each occupying distinct trophic and spatial niches (Ma et al., 2014; Wang et al., 2015; Zhou, 2014). Previous research has indicated that *S. oconnori* primarily consumes attached algae, such as diatoms, which are characterized by sharp, horny jaws and long gut lengths (Ma et al., 2014). In contrast, *S. waltoni* predominantly feeds on macroinvertebrates, which feature a cuspidal snout and shorter gut length (Zhou, 2014). Our observations revealed that the snout shape, gut length, and gill rake number of the natural hybrids were between those of *S. oconnori* and *S. waltoni*.

Ecomorphological associations are most readily identified in closely related species due to their extensive history of evolution and radiation in the same region (Zhang et al., 2008). Wang et al. (2015) explored the relationships between morphology, dietary habits, and spatial distribution in three *Rhinogobio* fishes and found that analyses integrating morphological variations contribute significantly to our understanding of the ecological niche within fish communities. Morphological analyses across different species aid in inferring ecological functions and exploring nutritional and spatial niches (Sampaio et al., 2013; Wang et al., 2015). Specifically, mouth size and gut length are associated with food size and food type, while snout whisker length, eye position, and eye size are related to the vertical positioning of food within the water column (Wang et al., 2015; Zhang et al., 2008). Hence, the emergence of natural hybrids between *S. oconnori* and *S. waltoni* may represent an evolutionary adaptation to the differentiation of nutritional and spatial niches in the middle Yarlung Zangbo River. Our understanding of the plateau adaptability of schizothoracine fishes will be improved by more research into the mechanisms of hybridization evolution, particularly in terms of cytonuclear genes combined with ecological habits. This research will also aid in the development of population management strategies on the Tibetan Plateau.

## AUTHOR CONTRIBUTIONS

**Baoshan Ma:** Conceptualization; methodology; software; writing – original draft. **Tianyi Zhao:** Data curation; investigation. **Bin Xu:** Writing – review and editing. **Liqiao Zhong:** Methodology; software. **Xiangxiang Wu:** Project administration. **Kaijin Wei:** Supervision. **Zhiming Zhang:** Writing – review and editing. **Yunfeng Li:** Funding acquisition.

## CONFLICT OF INTEREST STATEMENT

The authors declare there is no conflict of interest.

## Supporting information


Table S1.
Table S2.

## Data Availability

The data that support the findings of this study are openly available in the Dryad Digital Repository at https://doi.org/10.5061/dryad.xpnvx0kp4.
