# Supplementary material for: Morphological variation in Schizothorax oconnori, Schizothorax waltoni (Teleostei: Cyprinidae: Schizothoracinae), and their natural hybrids from the middle Yarlung Zangbo River, Tibet
Source: Ecol Evol. 2024 May 23;14(5):e11342. doi: 10.1002/ece3.11342 (PMC11116763; doi:10.1002/ece3.11342)
Supplement: Supplementary file 1 — Table S1. Table S2. [file ECE3-14-e11342-s001.docx]

**Table S1** Loadings of principal components for morphometric characters

|  | Component | | | |
| --- | --- | --- | --- | --- |
|  | 1 | 2 | 3 | 4 |
| Standard deviation | 7.771 | 4.079 | 2.15 | 1.121 |
| Proportion of variance (%) | 38.855 | 20.396 | 10.75 | 5.604 |
| Cumulative proportion (%) | 38.855 | 59.251 | 70.001 | 75.605 |

**Table S2** Loadings of principal components for truss distances

|  | Component | | | | |
| --- | --- | --- | --- | --- | --- |
|  | 1 | 2 | 3 | 4 | 5 |
| Standard deviation | 10.588 | 4.420 | 2.252 | 1.393 | 1.270 |
| Proportion of variance (%) | 42.351 | 17.678 | 9.006 | 5.572 | 5.080 |
| Cumulative proportion (%) | 42.351 | 60.029 | 69.036 | 74.607 | 79.687 |
